# Practical issues concerning tear protein assays in dry eye

**DOI:** 10.1186/s40662-014-0006-y

**Published:** 2014-11-13

**Authors:** Sharon D’Souza, Louis Tong

**Affiliations:** Narayana Nethralaya Superspeciality Eye Hospital and Post Graduate Institute, Bangalore, Karnataka India; Singapore Eye Research Institute, 11, Third Hospital Avenue, Singapore, 168751 Singapore; Singapore National Eye Center, Singapore, Singapore; Duke-NUS Graduate Medical School, Singapore, Singapore; Yong Loo Lin School of Medicine, National University of Singapore, Singapore, Singapore

**Keywords:** Human, Dry eye, Review, Tear, Proteomics, Biomarker

## Abstract

Dry eye is a common clinical condition diagnosed by cumulative evidence of symptoms and signs. Many new treatments in dry eye are either expensive, invasive, have potential for side effects, or are not easily accessible. In severe dry eye, the ideal modality of treatment to begin with is often not clear as specific molecular disturbances are not evident from just examination of clinical manifestations. Assessing the effects of ongoing treatment is not straight forward since there is lack of agreement between clinical signs and symptoms. There is a need to have more objective methods of selecting treatment for dry eye and monitoring the effect of treatment.

Recently, there are many new technologies applied to the discovery of tear biomarkers, for e.g., mass spectrometry based proteomics techniques and multiplex assays such as the bead-based sandwich indirect immunofluorescent assays. Tear proteins assays have even been made available as point-of-care devices. This review focuses on the evidence for the involvements of tear proteins in dry eye, possible changes in tear concentrations with therapy and the strength of evidence regarding dry eye pathology. Much remains to be done in terms of developing office-based assays and ascertaining their reliability, but current evidence suggests that tear proteins have a role in the clinical practice of dry eye.

## Introduction

### Diagnosis of dry eye

According to the International Dry Eye Workshop, “dry eye is a multifactorial disease of the tear and ocular surface that results in symptoms of discomfort, visual disturbance, and tear instability with potential damage to the ocular surface. It is accompanied by increased osmolarity of the tear and inflammation of the ocular surface” [1]. Simply put, dry eye is a complex multifactorial disorder affected by pathological processes such as lacrimal gland inflammation [2], meibomian gland dysfunction [3], and tear hyperosmolarity [1].

The signs and symptoms of dry eye disease do not always correlate well even though both are important in the diagnosis and management of dry eye. There are a number of tests used in clinical practice for the diagnosis of dry eye including the traditional Schirmer test, tear break up time, rose bengal staining and the more advanced technologies like the tear osmolarity, meibography and tear film interferometry. Unfortunately the available diagnostic tests often do not correlate reliably with severity of the patient’s symptoms [4].

Recently, a panel of tear proteins has been found, which may be specific and sensitive for the detection of dry eye [5]. A similar panel has been advocated for the diagnosis of primary Sjogren syndrome [6]. The importance of this discovery lies in the fact that most of the diagnostic tests need to be used in combination to reliably diagnose dry eye. These tear proteins may thus prove invaluable to the practicing ophthalmologist. Disease specific signatures of tear proteins may demonstrate underlying disease pathways, and about 4-5% of proteins may be deranged in dry eye [7]. Since tear fluid has various components secreted by different glands, the composition of tears reflects the health of different components of the ocular surface [8]. For some time now, the level of secretory IgA in the tears was used as a measure of lacrimal function, and could be used as a marker of ocular surface inflammation. It fluctuates diurnally in normal people, with a higher value at noon time, and has also been found to be reduced in dry eye compared to controls [9].

What is the cause of increased cytokines in dry eye? It is hypothesised that in the dry eye, the increased concentration of cytokines is not due to evaporation, but rather attributed to the upregulation of inflammatory genes in the conjunctival epithelium. This results in the increased production of inflammatory cytokines in tears [10]. The increase in tear cytokines was correlated to derangements in tear function tests such as osmolarity [11], suggesting that they reflect the underlying disease mechanisms.

In fact when a neural network was used to examine the tear proteins of dry eye patients and normal participants, multivariate analysis was able to discriminate the proteins from these two groups [12]. Specific protein derangements in dry eye patients further point to clinical subgroups of patients such as meibomian gland dysfunction [13], preservative induced dry eye in glaucoma patients [14], aqueous deficient dry eye [11], or diabetes [15].

### Differential diagnosis

The symptoms of dry eye can sometimes be non-specific. Dry eye symptoms like chronic irritation, tearing and eye redness may be mimicked by other conditions like allergic conjunctivitis [16]. Therefore, an objective test would be useful to distinguish dry eye from its mimickers. In fact, a decrease in tear break up time, which is a hallmark of certain types of dry eye disease, when present in isolation without the other signs of dry eye, may be a feature of allergic conjunctivitis [17], thus suggesting that it is unreliable to diagnose dry eye with only one single clinical test. In patients with chronic conjunctivitis, an elevated tear IgE may be a clue to underlying allergic conjunctivitis [18]. Cytokines in dry eye involve mainly Th1 and Th17 subsets whereas in allergic eye disease, T helper type 2-related mechanisms are involved in the sensitization phase, but both T helper type 1 and type 2 cytokines are overexpressed in the active disease, contributing to the development of ocular inflammation [19].

Other conditions we need to differentiate from idiopathic dry eye are those in which dry eye is associated with an underlying systemic cause. It may be possible to have a higher index of suspicion for a systemic cause of dry eye based on the levels of specific tear glycoproteins in addition to relevant history and clinical findings. For example, conditions such as pemphigoid may affect the glycoproteins differently from age-related dry eye [20].

It is important to assess the suitability of a tear protein as a biomarker before analysis. In this article we consider a tear protein to be potentially suitable as a clinical biomarker if it fulfils the following three criteria in the literature available: Firstly, the tear proteins which have been studied in dry eye disease in humans and found to be consistently deranged with a clearly defined normal range may be considered to be more relevant for clinical use. Secondly, the tear proteins that have been shown to change with the severity of dry eye, especially in longitudinal studies or trials are given importance. Lastly, there should be some link between dry eye pathology and the function of the measured tear protein. Such a link can be provided by relevant in vivo experiments in animals or an intervention that produced a known biological effect such as inflammation in humans.

The factors which we used to assess the suitability for use as clinical markers are shown in Table 1. For simplicity, we only show one member of each class or type of protein (except the class called ‘lacrimal proteins’). These interpretations are based on subjective evaluation of the current evidence on the tear protein studies presented in this review, and may need to be revised when new studies are published (Table 1). There is no published review which focused on these 3 criteria for determining the clinical relevance of the tear proteins in dry eye, therefore, we aim to provide a concise guide to enumerate these factors for the common tear proteins.

**Table 1:** **Evidence for assessing the suitability of tear proteins for clinical use**

Class of tear protein	Potential clinical marker	Human tear levels show consistent dysregulation	Human tear levels linked to clinical signs/symptoms of dry eye	Change with treatment or severity of dry eye?	Biological function in dry eye pathology known?
Lacrimal protein	Lactoferrin	++++ [21]-[25]	+++ [26],[27]	+ [28]	++ [29]
Protease	MMP-9	+++ [30]-[32]	+++ [11]	+++ [33]	+++ [34]-[42]
Lacrimal protein	Lysozyme	++ [21],[43]-[45]	+ [43]	0	0
Mucins	MUC5AC	+++ [46],[47]	+ [48]	0	++ [49],[50]
Lipid binding protein	Lipocalin	++ [45],[51]	+ [52]	++ [52]	+ [53]-[55]
Interleukines	IL-6	+++ [10],[31],[56],[57]	+ [58]	++ [58]	+ [59]-[61]
Chemokine	IL-8	++ [10],[57],[62],[63]	++ [62]	0	++ [61],[64]
Keratinisation-related	S100A8/9	+++ [5],[13],[14],[65]	+++ [5],[13]	0	+ [66],[67]
Epithelial health	EGF	++ [57],[64],[68]	+ [69]	0	++ [70]
Neurotrophic health	NGF	+++ [71],[72]	++ [71],[73],[74]	++ [73],[74]	++ [75],[76]

++++ strong evidence.

+++ good evidence.

++ modest evidence, some uncertainties about implication.

+ some evidence but studies may have conflicting results.

0 no clear evidence.

## Review

### Tear proteins

#### Available technologies and use in dry eye

Traditional separation of tear proteins was performed by electrophoresis [77], and identification and quantification by the enzyme linked immunosorbent (ELISA) method [78], but the recent advances in analytical technologies have provided ocular surface scientists with a host of other techniques for examination of tear proteins. The nano-scale sensitivity of some recent technologies enable the detection of proteins in tears from individuals rather than pooled samples [79],[80]. Some of the techniques described below can be used for relative or even absolute quantification if standards of known concentration are also available for calibration. One of the techniques of separating proteins rely on high performance liquid chromatography [81], or nano-electrospray liquid chromatography [82], where the size and charge of proteins provide the basis for their separation. The various techniques available are isobaric tagging using relative and absolute quantification (iTRAQ) technology, and the matrix assisted laser desorption ionisation (MALDI) time of flight (TOF) technique with mass spectrometry [83]. A variant of this technique is the surface-enhanced laser desorption/ionization (SELDI) TOF which can also be coupled with mass spectrometry [65].

Two dimensional (2D) separation of tear proteins based on mass/charge followed by dye staining have also been performed for separation and later identification of tear proteins [84]. In diabetic people, tear proteins have been evaluated using the 2D electrophoresis technology [85]. Isoelectric focusing in combination with sodium dodecyl sulphate (SDS) polyacrylamide gel electrophoresis (PAGE), an older technology, was able to detect glycation at specific residues of tear proteins such as lactoferrin and immunoglobulins [86].

Protein arrays have been used to detect proteins from tears of Sjogren syndrome patients [87]. This involves the binding of tear proteins to a predetermined set of antibodies arranged in a fixed arrangement in a stationary platform. In some unique scenarios, for example, to compare the types of immunoglobulins in tears, a more traditional assay such as the Western or immunoblot assay can be performed [88]. For office-based diagnosis, tear proteins can be separated in a very short time using a commercially available equipment, the Agilent Bioanalyser 2100, and analysed using the microfluidic based lab-on-chip technology [89]. For the analysis of a dozen or more cytokines and chemokines in a few microliter of tears, it seems the best technology is the multiplex bead-based immunofluorescent sandwich assay, and the sensitivity for detection of some cytokines are in the order of several picogram/milliliter concentrations, surpassing even the minimum detection limits of mass spectrometry based methods [90].

### Limitations

There are some pitfalls to tear protein analysis such as: differences in methods of tear collection which can give different results [91]. Tear collection may be done using Schirmer strips, capillary tubes, or even a special minisponge [92]. A key consideration is the comfort and minimal stimulation of the patient during tear collection, avoiding reflex tearing, since reflex tears likely have different tear protein levels from basal tears. Tear concentrations of interleukin (IL)-1 alpha, precursor IL-1 beta, and IL-1 receptor antagonist (RA) may be altered in dry eye in the basal but not the reflex tear [30]. The Schirmer test method has been shown to be reliable for the collection of tears for analysis of multiple cytokines [93], and such evaluation would be necessary as a prerequisite for new methods of tear collection proposed in the future.

The techniques of iTRAQ, MALDI TOF, SELDI TOF and 2D electrophoresis have shown to be useful for evaluation of tear proteins in a research setting but are unlikely to be used in clinics.

In summary, there is no universal methodology for handling or analysing tear proteins that is applicable to all clinics. Clinicians should consider the advantages and pitfalls of various methods should they want to apply these in their practice. Once applied, the same method should be continued for subsequent encounters and other patients.

### A ‘traditional’ marker: Lysozyme

This protein, also known as muramidase or N-acetylmuramide glycanhydrolase is a glycoside hydrolase, which are enzymes that damage bacterial cell walls by catalyzing the hydrolysis of 1,4-beta-linkages between N-acetylmuramic acid and N-acetyl-D-glucosamine residues in a peptidoglycan. Lysozymes are abundant in a number of secretions, such as tears, saliva, human milk, and mucus [94]. Lysozyme is probably the earliest tear protein studied in conjunction with dry eye, and the level of tear lysozyme was noted to increase with age till 40 and decrease thereafter [43]. It was found to be decreased in glaucoma patients with chronic medication induced dry eye [44]. In a large study with 262 patients (78 patients with Sjogren syndrome), the concentrations of the lysozyme and lactoferrin protein in tear samples in Sjogren syndrome were determined [21]. It was found that lysozyme was decreased in idiopathic dry eye and Sjogren syndrome compared to controls [43]. However, a later study showed that lysozyme concentration did not differ between non-Sjogren dry eye, Sjogren syndrome or normal controls [45]. Researchers also found the single protein lysozyme test to be insufficient for the diagnosis of dry eye [95]. Nevertheless, lysozyme levels may be useful in specific contexts, for example, to check for adverse effects of beta-adrenergic receptor blocking drugs such as Practalol, which reduced tear lysozyme levels [96] or to detect dry eye from specific occupational exposures in coal mining [97]. With so many existing and emerging technologies, it may be worthwhile to take a fresh look at the utility of lysozyme.

### Lactoferrin revisited

Lactoferrin, also known as lactotransferrin, is a non-haem iron-binding protein of the transferrin family. It is a globular glycoprotein with a molecular mass of about 80 kDa that is widely represented in various secretory fluids, such as milk, saliva, tears and nasal secretions. Lactoferrin is one of the components of the immune system of the body. It has antimicrobial activity, is antiviral, antiparasitic, catalytic, immunomodulatory and anti-inflammatory [98].

It was found to be negatively correlated to Rose Bengal staining, indicating that reduced lactoferrin was a marker of ocular surface damage [26]. However, in evaporative dry eye in the absence of epithelial defects, tear lactoferrin was also found to be reduced [99]. In chronic hepatitis C patients, the cotton thread test of tear secretion was weakly correlated (r = 0.35) to the tear lactoferrin levels [27], indicating that these patients having significantly lower tear lactoferrin levels than control participants. The use of tear lactoferrin has been advocated for the diagnosis of primary Sjogren’s syndrome, where the test had a specificity of 95% and a sensitivity of 72% [22]. These values were somewhat superior to using only the Schirmer I test for detection of this disease [22]. Researchers have suggested a cut-off value of 1.1 mg/mL for tear lactoferrin so that the assay has optimal accuracy for the diagnosis of dry eye [23]. Using this threshold, the test was able to detect dry eye with a sensitivity 79.4% and specificity of 78.3% [24]. Using a convenient commercial lactoplate assay, the tear lactoferrin was measured in dry eye patients and was decreased relative to controls [25].

However, it has been found that lactoferrin changes either do not appear early enough for diagnosis of mild to moderate dry eye or that some cases of dry eye did not have the lacrimal dysfunction that this assay tested for [100]. In any case, treatment of dry eye with punctal occlusion was associated with increased tear lactoferrin levels, suggesting that tear levels of this protein may be a measure of tear turnover [28]. Recently a TearScan™ Lactoferrin Diagnostic Test Kit was made available commercially (Advanced Tear Diagnostics, Raleigh, NC). This device provides quantitative point-of-care tear concentration of lactoferrin.

### S-100A proteins

The S-100A protein is a family of low molecular weight proteins characterized by two calcium binding sites of the helix-loop-helix conformation and at least 21 different types of S100 proteins are known. It is normally present in cells derived from the neural crest (Schwann cells, melanocytes and glial cells), chondrocytes, adipocytes, myoepithelial cells, macrophages, Langerhans cells, dendritic cells, and keratinocytes. S100 proteins are involved in the regulation of protein phosphorylation, transcription factors, calcium homeostasis, the dynamics of cytoskeleton constituents, enzyme activities, cell growth and differentiation, and the inflammatory response [101].

The S100 A8 and A9 proteins have been known to be pro-inflammatory [66]. In a study using the isobaric tagging for relative and absolute quantification (iTRAQ) technology, we found the S100A8 and S100A9 proteins were among a panel of 6 upregulated proteins found in the tear of dry eye patients [5]. The average ratio of S100A8 and S100A9 in dry eye versus control subjects was 1.82 (SD 1.41) and 1.92 (SD 1.48) respectively. In another study using a different SELDI-TOF technique, over-expression of S100A8 has also been reported [65].

### Mucin and related molecules

The mucins (MUC) are a family of high molecular weight, hydrophilic, heavily glycosylated proteins, produced by epithelial tissues. The tear film on the ocular surface epithelia is maintained by the mucins on its surface as well as by membrane-associated mucins in the apical surface of the cell. They are secreted by goblet cells or other secretory cells and have a characteristic ability to form gels with the exception of the monomeric MUC7, hence they have various functions from lubrication to cell signalling to form chemical barriers. MUCs 1, 3A, 3B, 4, 12, 13, 15, 16, 17 and 20 are membrane associated and MUCs 2, 5 AC, 5B, 6, 7 and 19 have been classified as secreted mucins [102].

Using Schirmer strip samples, mean MUC5AC content in tears was found to be lower in the dry eye patients than in the age- and gender-matched healthy individuals [46]. The levels of certain mucin molecules were associated with certain ocular surface states. For example, tear MUC5AC was reduced in symptomatic contact lens wearers, and MUC4 was correlated to the presence of temporal lid parallel conjunctival folds (conjunctivochalasis) and lid wiper epitheliopathy [48]. The levels of mucins are not always depressed in dry eye. Sjogren syndrome patients exhibited increased soluble MUC16 in the tear compared to controls [47]. The membrane-associated MUC16 and the mucin-associated T-antigen carbohydrate were associated with ocular surface epithelial protection [103].

Understanding of tear mucin regulation may produce insight into the mechanism of at least some types of dry eye [49]. Phospholipid transfer protein, a protein that interacts with mucins, may also be relevant to the dry eye mechanism [104], but this has not been studied well enough to be used for clinical purposes. Since the regulation of mucin affects dry eye pathologic mechanisms, it gives greater credibility to the use of mucins as a marker for this disease.

### Proteases

Protease refers to a group of enzymes whose catalytic function is to hydrolyse (breakdown) peptide bonds of proteins. They are also called proteolytic enzymes or proteinases. Proteases differ in their ability to hydrolyse various peptide bonds. Matrix metalloproteinases (MMPs) are a class of proteases belonging to the metzincin superfamily characterised by zinc cofactors, and they are the most important proteases found in tears [105],[106].

These enzymes degrade extracellular matrix proteins, but can also process certain bioactive molecules. They are also associated with cleavage of cell surface receptors, the release of apoptotic ligands (like the FAS ligand), and chemokine/cytokine in/activation. MMPs also play a major role cell proliferation, migration (adhesion/dispersion), differentiation, apoptosis, and host defense [106]. MMP-9, also known as 92 kDa type IV collagenase or gelatinase B, can be involved in the degradation of collagen IV present in the basement membrane and extracellular matrix.

In rosacea-associated meibomian gland disease or Sjogren’s syndrome, the tear activity of MMP9 was raised compared to controls [30]. In another study, pro-MMP-9 levels were found to be significantly elevated in various ocular surface diseases: blepharitis (p = 0.013), allergic eye disease, dry eye and conjunctivochalasis (all p < 0.001) compared to controls [31]. In a study involving 46 patients with newly diagnosed dry eye and 18 control participants, tear MMP-9 activity was assessed with an MMP-9 activity assay in 1 uL of basal tear fluid [32]. The MMP-9 activity in the control group was 8.39 +/- 4.70 ng/mL and progressively higher levels of MMP-9 were found in the dry eye groups with the highest levels corresponding to the most severe dry eyes clinically [32]. Although this is not a longitudinal study, it represents correlation of MMP-9 levels with clinical disease severity and suggests that it can be a clinical marker for monitoring patients.

In a study involving treatment of ocular surface disease, including dry eye, with a therapeutic contact lens, clinical improvement was observed and at the same time, tear MMP-9 was found to decrease by day 7 and further decrease to minimal levels by day 21 [33]. These types of longitudinal results strengthen the validity of using the tear MMP-9 assay as a monitoring tool.

InflammaDry (RPSInc, Sarasota, FL, USA) is a rapid (10 minute) point of care diagnostic test for tear MMP-9, which gives a positive result if tear MMP-9 exceeds 40 ng/ml. This test can be easily administered by a nurse or technician. It has a sensitivity of 87% and specificity of 92% (RPS clinical study: protocol number 09-001, version no 2.4) when compared to a combined diagnostic criteria of dry eye tests (OSDI > =13, Schirmer II < 10 mm, TBUT < 10 sec and staining > =1). This disposable test kit is based on direct sampling of tears from the inferior fornix using microfiltration and capturing of MMP-9 between specific monoclonal and polyclonal antibodies, and does not require specialised equipment. However, in its current form, the InflammaDry does not yield the actual MMP-9 concentration [107].

Experimentally, dry eye models in animals have demonstrated the importance of tear MMPs in the induction of ocular surface damage in dry eyes [34],[35] and have linked these to inflammatory signaling [36], thereby strengthening the biological basis for clinical use of MMPs. In experimental dry eye, the raised MMP-9 can also be detected in the cornea and lacrimal tissues, implying that tear concentrations were not raised purely due to evaporation [37]. In primary lacrimal gland cultures, pro-MMPs were secreted into the culture medium, suggesting that the lacrimal gland may be a source of MMPs [38].

### Lipocalin

Lipocalins are a group of extracellular low molecular weight proteins [108], which use multiple recognition properties including ligand binding to small hydrophobic molecules, macro-molecular complexes and binding to specific cell surface receptors. Tear lipocalin is a major protein in tears, which binds a variety of lipophilic molecules. It can also bind to macromolecules like lactoferrin and lysozyme and has a variety of functions in tears, including anti-inflammatory activity, binding and release of lipids [53], regulation of tear viscosity, endonuclease inactivation of viral DNA and used as a biomarker for dry eye [109].

In dry eye disease, downregulation of lipophilin-1 and lipocalin-1 have been found [99]. In fact, the tear lipocalin was even lower in Sjogren’s syndrome patients compared to non-Sjogren’s dry eye patients [45]. In a treatment trial involving dry eye volunteers, improvement of clinical signs of dry eye with an increase in the stability of tear film was found in conjunction with an increase in the tear lipocalin levels [52]. One risk factor for dry eye is contact lens wear, and dry eye sufferers also tend to have intolerance to contact lens. Surprisingly, people who are intolerant of contact lens wear demonstrated *higher* tear lipocalin levels compared to people who tolerated contact lens [110], suggesting that tear lipocalin alone should not be used for the diagnosis of dry eye. To the best of our knowledge, there has been no longitudinal clinical study on levels of lipocalin to-date.

Experimentally, rabbit levels of tear lipocalin were hormone dependent. Ovariectomy in rabbits decreased tear lipocalin and in contrast, administration of estrogen or male androgen dihydrotestosterone (DHT) increased the levels [54]. These studies point out that hormones act upstream of the production of lipocalin. Interestingly, addition of exogenous sex steroids resulted in the binding of these steroids to the tear lipocalin [55]. Since levels of hormones influence the human dry eye, these findings provide some evidence linking lipocalins to the biology of dry eye. Unfortunately, there has been no evidence of tear lipocalin deficiencies in animal models of dry eye so far.

### Interleukines

The interleukins (IL), a group of cytokines that were first seen to be expressed by leukocytes, have an important role in the adaptive immune response in that they are required for the propagation of inflammation [111]. Recently, a panel of four key inflammatory cytokines (IL-1β, IL-6, IFN-γ, and TNF-α) were found to be highly reproducible and reliable when determined in tear samples as little as 4-10 μL. Standard operating instructions for tear collection, shipping, storage and processing were recommended [112].

### Interleukin 1

Tear ILs, in particular IL-1β has been shown to increase in aqueous deficiency dry eye [113].

In patients with meibomian gland dysfunction MGD and those with Sjogren’s syndrome, compared with normal participants, the concentrations of tear IL-1α and mature IL-1β were increased, and precursor IL-1β was decreased [30]. Experimentally, the production of IL-1β in tears has been linked to inflammation [39],[40],[59],[60],[114],[115].

### Interleukin 6

Tear IL-6 has been found to be elevated in Sjogren’s and non-Sjogren syndrome dry eye patients [56],[57]. In another study, significant elevation of tear IL-6 has also been observed after 2 weeks of soft contact lens wear. Since the tears break up time and expression of MUC5AC was also decreased over this time, the study suggests that increased tear IL-6 may be an indicator of worsening epithelial and mucoid function [58]. Interestingly, one of the polymorphisms in the IL-6 gene was associated with dry eye disease [116]. This not only suggests a genetic predisposition of dry eye for certain populations, but also supports the use of IL-6 as a marker of dry eye in susceptible people.

### Interleukin 17

The tear concentrations of IL-17 in patients with filamentary keratitis, graft versus host disease, autoimmune keratitis, Sjogren’s syndrome, dry eyes, MGD and Steven Johnson syndrome were significantly higher than in normal study participants [117]. This relationship of increased tear IL-17 was only observed in patients with systemic autoimmune disease but not in those whose inflammation is restricted to the eye. Interestingly, serum IL-17 levels were correlated to fluorescein staining scores, but this study did not investigate tear IL-17 levels [118].

### Tumour necrosis factor-α (TNF-α) and Interferon-γ (IFN-γ)

These are both acute response cytokines that enhance cellular immune responses [119]. Although tear TNF-α levels were higher in dry eye than controls [57], there was no significant correlation between these levels and the dry eye clinical tests [56]. There was also no difference between the tear TNF-α levels between Sjogren’s and non-Sjogren’s dry eye patients [56]. The pro-inflammatory role of tear TNF-α however, has been verified in experimental models [39],[59],[60],[120],[121]. The main drawback in the use of tear cytokines as markers is that none of these have been measured longitudinally in clinical studies to-date.

Tear levels of IFN-γ were elevated in Sjogren’s syndrome compared to controls [62]. Similarly, the tear levels of IFN-γ in cystic fibrosis, a systemic disease associated with dry eye, were significantly higher than those in non-cystic fibrosis controls [63],[122]. As in the case of TNF-α, the pro-inflammatory role of IFN-γ has been verified in experimental murine dry eye [121],[123].

### Chemokines and interleukin 8 (CXCL8)

The tear chemokines which have been implicated in dry eye are CX3CL1 [64], CXCL10 [64], CCL4/MIP-1beta [124],[125], CCL3/MIP-1alpha [57],[125],[126], CCL5/RANTES [57], CXCL9, -10, and -11 [127]. The tear levels of CXCL9, -10 and -11 were 1,148 +/- 1,088, 24,338 +/- 8,706, and 853 +/- 334 pg/mL, in dry eye, and only 272 +/- 269, 18,149 +/- 5,266, and 486 +/- 175 pg/mL in controls respectively [41]. Hyperosmolarity induced the production of Monocyte chemotactic protein ( MCP)-1 in experimental (epithelial cell culture) settings, providing a link between tear chemokines and dry eye pathology [61]. Nevertheless, there is currently no evidence that chemokines are reduced after treatment of dry eye or that measurement of chemokines is specific and sensitive for the detection of dry eye.

The tear levels of proinflammatory cytokine IL-8 was found to be higher in Sjogren’s syndrome compared to controls [62]. The levels were also higher in dry eye with and without MGD compared to normal controls [57]. In experimental dry eye, IL-8 was also found to be upregulated in the lacrimal gland [36],[37]. Ocular pain levels were correlated to tear IL-8 [64].

### Growth factors and wound healing molecules

A number of growth factors are produced by the lacrimal gland/ocular surface and may provide trophic effects for ocular surface epithelium. Intuitively, the levels of tear growth factors may reflect the extent of trophic support that is vital for epithelial health [128].

### Nerve growth factor and related proteins

It is logical to assess proteins related to nerve endings in dry eye, since dry eye may result from an interruption of neural reflex at the afferent nerve endings. The tear nerve growth factor (NGF) levels were higher in dry eye patients compared to age- and gender-matched healthy control participants. In these patients, prednisolone treatment for 28 days resulted in a decrease in tear NGF levels, which occurred together with clinical improvement of dry eyes [73]. In another study, tear levels of NGF were increased in dry eye patients whereas related peptides: Calcitonic Gene Related Peptide (CGRP) and Neuropeptide (NP) Y concentrations were decreased compared to healthy participants. Furthermore, the level of tear NGF was correlated with clinical severity of dry eye while CGRP and NPY levels were inversely correlated to these clinical parameters [71]. Tear NGF/total tear protein ratio was increased in photorefractive keratectomy and laser in situ keratomileusis, and the early post-operative levels were also correlated with tear function 6 months later [74]. NGF was elevated in tears of contact lens wearers with dry eye, and the levels were associated with a decrease in the nerve plexus density in the cornea [72], supporting the theory that dry eye results from a decrease in the afferent part of the lacrimal loop.

The tear concentration of another peptide, substance P, was also associated with dry eye, and was elevated after excimer corneal surgery compared to pre-surgical levels [129].

Is there any evidence of functional mechanisms involving neural peptides? Experimentally, NGF has been shown to induce goblet differentiation and increase in MUC5AC both in human conjunctival epithelial cells exposed to increasing NGF concentrations and confirmed in primary cultures [75]. Dry eye was induced in rats by subcutaneous scopolamine treatment and aqueous tear production, tear clearance, fluorescein corneal staining, and tear break-up time were evaluated. The NGF mimetic was able to induce an improvement of dry eye test parameters and glycoprotein secretion [76]. The use of supplementary NGF for dry eye therapy is however, hard to justify if the tear level of this protein is already higher than normal to start with. Further studies are required to determine why the NGF levels are elevated in dry eye, and perhaps the use of NGF as a biomarker should be delayed until substantial longitudinal studies of NGF levels are available.

### Other growth factors

Epidermal growth factor (EGF) is a low molecular weight polypeptide that acts by binding with high affinity to the epidermal growth factor receptor (EGFR) on the cell surface. Stimulation of the intrinsic protein-tyrosine kinase activity of the receptor ultimately leads to DNA synthesis and cellular proliferation, differentiation, and survival [130]. The concentration of tear EGF was significantly decreased in non-Sjogren dry eye, Sjogren syndrome, and Steven Johnson syndrome patients compared with controls [68]. On the other hand, higher levels of tear EGF has been linked to subepithelial fibrosis in dry eye [69]. In animal models, addition of EGF has been linked to longer tear break up times and lower fluorescein staining scores, establishing a functional effect of this growth factor in the ocular surface [70].

Transforming growth factor (TGF) is a peptide known to be involved in inflammatory and fibrotic pathways. TGF-β1 is the prototypic member of the transforming growth factor superfamily and it elicits diverse cellular responses like proliferation, induction and regulation depending on cell type, state of differentiation and culture conditions [131]. In dry eye, tear TGF-β bioactivity, as assessed by a cell based assay was found to be higher (9777.5 +/- 10481.9 pg/mL) than those in controls (4129.3 +/- 1342.9 pg/mL). The level of TGF-β bioactivity was highest in those with Sjogren syndrome, compared to controls and non-Sjogren dry eye [132]. The biological role of TGF-β has also been investigated in experimental dry eye settings [36],[37].

Corneal neovascularisation may be observed in very advanced dry eye and ocular surface failure [133]. Vascular Endothelial Growth Factor (VEGF) is a signal protein that stimulates vasculogenesis and angiogenesis, cell survival, migration and differentiation. When VEGF is overexpressed, it can contribute to neovascularisation [134]. Tear VEGF levels have been found to be raised in dry eye compared to controls in a study comparing evaporative dry eye to normal subjects. Epidermal growth factor (EGF), fractalkine/CX3CL1, IL-1-receptor antagonist (RA), IL-8/CXCL8, interferon inducible protein (IP)-10/CXCL10 were found elevated along with VEGF in 94%–100% of samples [64]. Despite the promise of using growth factors as tear biomarkers, there has yet been no longitudinal study that evaluated the tear levels of these proteins.

### Miscellaneous tear proteins and future directions

There are some interesting proteins that have not been evaluated as thoroughly as those mentioned above, but future research will show their usefulness or otherwise in clinical scenarios. The phospholipase (PL)A2-IIa concentration was found to be lower in patients with ocular rosacea (31.0+/-18.4 μg/ml) and in patients who had dry eye (25.8+/-15.1 μg/ml), compared to normal controls [135]. The activity of serum PLA2-IIa was significantly increased in tears from dry eye diseased patients compared to those from normal subjects. In addition, serum PLA2-IIa stimulated the production of prostaglandin E(2) in ocular surface epithelial cell cultures, linking this tear protein to inflammation [135]. We have previously reported the involvement of other proteins (e.g., prolactin-induced protein, enolase and orosomucoid) in dry eye [5]. These have not been as well characterized as the other tear proteins above. Lacritin is a tear protein that is reduced in dry eye. Because it protects against cells from stress, replacement of lacritin has been advocated as a form of treatment in dry eye. Furthermore, an ELISA assay has been developed to quantify lacritin levels [136].

Specific molecules such as B cell activating factor may indicate severity of inflammation in Sjogren syndrome [137]. Similarly, tear aquaporin 5 was increased in the tears of Sjogren syndrome, indicating the level of lacrimal damage [68]. Increased levels of anti-Ro or anti-La may also be detected in tears of Sjogren syndrome [138], although the role of this assay is currently not clear. The tear levels of pro-apoptotic proteins such as sFas have been measured in patients with dry eye associated with cystic fibrosis [139], and it is possible that subject to further studies, this marker may have some clinical application.

In addition, the tear levels of albumin may also serve as a marker of inflammation. In one study the levels of albumin were found to be increased in glaucoma patients with dry eye [44]. Some proteins are poorly understood and there is no clear strategy that can ‘restore’ this to normal. For example, we found the scaffolding protein 14-3-3 to be uniquely upregulated in glaucoma patients with dry eye who used chronic medications but not in other dry eye patients [14]. It may be possible to use such markers to monitor these patients’ progress even if the biological link is unclear, but more research such as longitudinal studies would be required to address this.

A great difficulty in diagnostic science is the wide variation of techniques used in analysis. The differences in techniques can result in the variation of the ‘normal’ range of specific tear proteins [140]. The extent of variation of tear proteins during processes such as fasting should also be further investigated [141]. Particular care should be paid to the conditions during tear collection: close eye tears and open eye tears differ in the fibronectin concentrations [23]. It may be necessary for an international panel to set up universal standards for the testing of specific tear proteins, or testing of patients for specific purposes, such as for the selection of treatment modality in dry eye.

Much remains to be done in terms of developing clinic based assays and ascertaining their reliability. Technologies that rapidly produce results and take up minimal amount of space are highly promising, such as the one using a microfluidics chip mentioned above [89].

Although this review focuses on proteins, tear lipids mediators in the tear may also have a major role in inflammation, and are potentially useful biomarkers. For example, the omega-3 fatty acid metabolic pathways produce both pro-inflammatory lipids (leukotrienes) as well as anti-inflammatory lipids (e.g., the 18R-, 17R- and 18S- resolvins). The resolvins (Rv)E1, neuroprotectin D1 and RvD1 dampen a range of immune responses including T cell responses, cytokine production, and endothelial adhesion. In fact, the RvE1 analog Rx-10045 has completed phase II testing for treatment of dry eye (clinicaltrials.gov NCT01675570). Assuming that diagnostic technology of lipid detection overcomes certain practical obstacles, tear lipids may be useful biomarkers for guiding therapies.

## Conclusions

We propose that when a patient presents with dry eye symptoms, the history and the clinical examination will alert to any systemic diseases that may be linked to dry eye, for example, rheumatoid arthritis or Sjogren’s syndrome. In recalcitrant cases of dry eye, tear proteins may be useful for selection of treatment or following up the patient for response to treatment. For example, increased tear MMPs may suggest the more prolonged use of MMP inhibitors like the doxycyclines. In conclusion, clinicians are reminded that tear protein science is an evolving area of research and with increasing knowledge on the pathogenesis of specific types of dry eyes, more appropriate biomarkers may replace these mentioned in this article.

### Retrieval of articles

The NCBI Pubmed database was searched for the keywords “Tear Proteins” and “Dry eye” in any field. This yielded 471 articles, and these have been manually curated to 131 relevant articles by excluding those that do not support the criteria in Table 1. Seven articles were added to introduce the proteins in each section.
